# Phenolic profile and antioxidant activity of free/bound phenolic compounds of sesame and properties of encapsulated nanoparticles in different wall materials

**DOI:** 10.1002/fsn3.2712

**Published:** 2022-01-09

**Authors:** Reza Esmaeilzadeh Kenari, Razie Razavi

**Affiliations:** ^1^ Department of Food Science and Technology Sari Agricultural Sciences and Natural Resources University Sari Iran

**Keywords:** controlled release, encapsulation, natural antioxidant, *Portulaca oleracea*, *Trigonella foenum‐graecum*

## Abstract

This study aimed to evaluate the antioxidant activity of free and bound flavonoid or phenolic compounds extracted from the sesame seed (Oltan and Yekta varieties) as natural antioxidants and to demonstrate the properties of nanoparticles. The total phenolic content (TPC) of Oltan was higher (864.70 mg GAE/100 g seed) than that of Yekta (629.23 mg GAE/100 g seed). Oltan took up higher amounts of free (516.86 mg GAE/100 g seed) and bound (347.83 mg GAE/100 g seed) phenolics than Yekta. Also, the Yekta variety exhibited lower amounts of free (45.89 mg CE/100 g seed) and bound flavonoids (21.51 mg CE/100 g seed) and the total flavonoid content (TFC) (67.40 mg CE/100 g seed). Chlorogenic acid was the major phenolic compound present in the sesame seed. In both the DPPH and ferric reducing antioxidant power (FRAP) assays, the highest antioxidant activity was observed in the Oltan variety. Free phenolics showed the highest antioxidant activity, followed by bound phenolics, free and bound flavonoids. Therefore, free phenolics of the Oltan variety were encapsulated in *Portulaca oleracea* and *Trigonella foenum‐graecum* seed gums. All nanoparticles showed nanometric size from 236.1 to 680.7 nm, negative zeta potential from −35.4 to −18.3 mV, high encapsulation efficiency from 61.35% to 74.49%, and desirable polydispersity index (PDI) between 0.315 and 0.332. Higher release of phenolics and sedimentation rate were observed in phenolic compounds encapsulated in Khorfeh and Shanbalileh, respectively. The gradual release of phenolic compounds, as well as sedimentation rate of composite coating during 40 days of storage, demonstrated that nanoencapsulated phenolics of sesame within the composite gum coating could be used as natural antioxidants in food systems.

## Graphical abstract

In this study, for the first time, the free/ bound phenolic compounds of sesame seed were extracted and then phenolic content, flavonoid content, and the antioxidant activity of different compounds were investigated. The results showed that free phenolics of sesame seed exhibited a higher antioxidant activity than bound compounds.

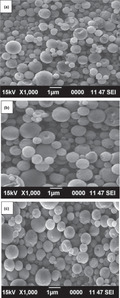

## 1 INTRODUCTION

The generation of free radicals is a continuous process that occurs as part of normal cellular function, but the excessive generation of these radicals is strongly implicated in many diseases (Dravie et al., 2020) such as cancer, diabetes, and heart and liver diseases. An alternative strategy for many of these diseases is extracting plant‐derived drugs to provide new functional ingredients with high bioactivity (Mekky et al., 2019) named antioxidant. Antioxidants scavenge reactive oxygen species to keep the balance between antioxidants and oxidants. They play a critical role in the prevention of numerous diseases (Lin et al., 2017) and food oxidation. Butylated hydroxytoluene (BHT), butylated hydroxyanisole (BHA), and tertiary butylhydroquinone (TBHQ) are the most consumed synthetic antioxidants (Şahin & Elhussein, 2018). However, their adverse effects on human health have led to their replacement by natural antioxidants for the application as biopharmaceutical, nutraceuticals, and food additives (Gharehbeglou et al., 2019). Therefore, there is a growing interest in the application of antioxidants derived from natural resources such as plant extracts (Şahin & Elhussein, 2018). Phenolics are metabolites broadly spread throughout the plants. They are potentially acting against the disease resulting from oxidative damage (Gharehbeglou et al., 2019). The elucidation of the bioactive compound's potential is a required step for future utilization of these compounds as antioxidant ingredients.

Sesame (*Sesamum indicum* L.) is a vital oilseed crop belonging to the family *Pedaliaceae* (Bodoira et al., 2017; Yazhen Chen et al., 2020; Lin et al., 2017). Sesame oil production ranks eighth in the world's oil market (Bodoira et al., 2017). Sesame seeds are beneficial for human health due to a rich mixture of phytochemicals such as phenolics, carotenoids, tocopherols, and phytosterols (Yazhen Chen et al., 2020; Lin et al., 2017). Products obtained from the extraction of sesame oil have already been used in both the pharmaceutical and food industries (Popović et al., 2020). Sesame seeds showed many biological activities such as antioxidant, anticancer, antidiabetic, and cardioprotective (Mekky et al., 2019). The presence of specific types of lignans, mainly sesamin, sesamolin, sesamol, and sesaminol, besides several phenolic compounds, is responsible for displaying critical biological properties (Bodoira et al., 2017). The quality and quantity of bioactive compounds of sesame seed depend on the physiological or ecological parameters such as climate, soil type, and variety (Dravie et al., 2020). The color of sesame seed exhibits a variation in different species, which could partly be attributed to changes in the composition of bioactive phenolics (Pathak et al., 2019). Therefore, to obtain extract rich in phenolic compounds and antioxidant activity, research is needed to determine the content of bioactive compounds and the antioxidant activity of various sesame. Today, new efforts are addressed to promote the nutraceutical value of foods in different ways. This includes obtaining encapsulated extract rich in bioactive compounds, which can be useful to the development of functional supplements and natural preservatives.

Phenolic compounds are known for their instability and sensibility to high temperature, pH, light, oxygen, and degradation enzymes (Escobar‐Avello et al., 2021). It is therefore important to perceive a strategy for protecting phenolic compounds, preserving their biological activities, and enhancing their bioaccessibility and bioavailability (Escobar‐Avello et al., 2021). Encapsulation of phenolic compounds with native seed gums is a new approach to overcome these drawbacks. Encapsulation is a technique in which bioactive compounds are encapsulated with coating materials or entrapped within carriers or shells (Garavand et al., 2021).

Recently, studies have extended our understanding of natural gums and their potential use in the food and pharma industries which has increased the application of natural gums (Dhull et al., 2020). *Portulaca oleracea* L., an annual herbaceous plant with reddish stems, is a valuable plant that belongs to the family Portulacaceae. In Persia, it is known as “Khorfeh” (Iranshahy et al., 2017). It is used as a traditional medicine to cure hypertension, coronary artery disease, cancer, gastrointestinal diseases, respiratory, insomnia, headache, and other inflammatory and anti‐immune disorders (Aye et al., 2020). *Trigonella foenum‐graecum* L. belongs to the family Leguminosae cultivated worldwide in many countries (Dhull et al., 2020). In Persia, it is known as “Shanbalileh.” It is recognized as a gelling and suspending agent, matrix former, and release retardant (Nayak & Hasnain, 2019). It has received particular interest due to its functional properties such as stability, foam formation, gelation, emulsification, and viscosity (Gadkari et al., 2018). The seeds of both plants produce a lot of mucilage, and when seeds are exposed to water, they swell and turn out to be slick. This study aimed to evaluate the antioxidant activity of free/bound flavonoid and phenolic compounds of the two varieties of sesame seed (Oltan and Yekta) and to demonstrate the potential use of different native seed gums as wall materials to encapsulate phenolic compounds of sesame seeds.

## 2 MATERIALS AND METHODS

### 2.1 Materials

The two varieties of sesame seeds, black (Oltan cultivar) and white (Yekta cultivar), were obtained from an agriculture farm in Mazandaran province, Sari, Iran. The obtained sesame seeds were stored in dark polyethylene bags at 4°C before use. Other chemicals were of analytical grade and sourced from Sigma Co. *Portulaca oleracea* and *Trigonella foenum‐graecum* seeds were purchased from the local market.

### 2.2 Methods

#### 2.2.1 Extraction of free flavonoid and phenolic compounds

Free flavonoid and phenolic compounds were extracted using the method described by Zhou et al. (2016). Briefly, 10 g of sesame seed samples was ground and then blended with 50 ml of n‐hexane for 1 min. After 3 min of centrifuging at 2700 *g*, the oil of pellet was extracted twice, and 10 ml of chilled acetone (80%) was added to the residue and stirred for 1 min. The supernatant was collected after double centrifugation at 2700 *g* for 3 min. Thereafter, it was dried in a vacuum oven at 45°C (Zhou et al., 2016).

To extract the bound flavonoid and phenolic compounds, the residue of the previous extraction stage was flushed with nitrogen gas, sealed, and digested with 20 ml of 4 M sodium hydroxide at 25°C for 90 min with shaking. Then, the mixture was acidified with HCl to pH 2.0 and extracted 10 times with ethyl acetate. Finally, it was dried in a vacuum oven at 45°C (Yongsheng Chen et al., 2015).

#### 2.2.2 Estimate the flavonoids and phenolic content

To measure the total flavonoid content (TFC) and the total phenolic content (TPC), the extract (1 g) was reconstructed with 10 ml of methanol (70%). The final concentration of the extract was 100 mg/L (Zhou et al., 2016). The TFC of sesame seeds was determined by the aluminum chloride colorimetric method. The sesame extract (1 ml) of each sample was mixed with 150 µl of 5% sodium nitrite solution (5% NaNO_2_) and rested for 5 min before the addition of 300 µl of 10% aluminum chloride (10% AlCl3). The resulting mixture was then allowed to stay for another 5 min before adding 1 ml of 1 M sodium chloride (1 M NaOH), and 1.05 ml of distilled water was vortexed for 10 s. The absorbance of the aliquot was measured against reagent blank at 510 nm using a UV spectrophotometer (TECAN Infinite M200 Pro), and the TFC was expressed as quercetin equivalent (QE) mg/100 g seed (Yazhen Chen et al., 2020). The TPC of sesame seeds was performed by the Folin–Ciocalteu method. An aliquot (100 µl) of each sesame extract was dissolved in 400 µl of distilled water and added to 100 µl of the Folin–Ciocalteu reagent for 6 min. Then, 1 ml of sodium carbonate 1 N was added and the mixture was incubated for 1 h at room temperature. The absorbance of the aliquot was measured against a blank reagent at 765 nm using a UV spectrophotometer (TECAN Infinite M200 Pro). The TPC was expressed as gallic acid equivalent (GAE) mg/100 g seed (Yazhen Chen et al., 2020).

#### 2.2.3 Phenolic compounds profile

The phenolic compound were analyzed using liquid chromatography systems (RP‐HPLC; Waters), equipped with a photodiode detector (M‐2998) and C18 column (4.6 mm 5 µ × 25 cm). To separate phenolic compounds, a mobile phase consisting 0.01% (v/v) acetic acid, acetonitrile (v/v) (solvent 1), and acidified water (0.1% (v/v) acetic acid) (solvent 2) was used. The constant flow rate was 0.5 ml/min. Methanol 0.1% (v/v) (solvent 2) was used to separate flavonoid compounds. The gradient and elution programs were described by Mekky et al. (2019).

#### 2.2.4 Antioxidant activity by DPPH free radical scavenging activity

The antioxidant activity of bioactive compounds was determined by the DPPH assay. Different concentrations (250, 500, and 1000 mg/L) of free/bound compounds (2 ml) were added to DPPH solution (0.1 mM), separately. After 30 min, the absorbance of the mixture was taken at 515 nm with a UV‐visible spectrophotometer at 4°C. The free radical scavenging activity was expressed as a percentage of scavenging activity (Ruslan et al., 2018).

#### 2.2.5 Antioxidant activity by ferric reducing antioxidant power

The FRAP assay was carried out according to a modified method of Ti et al. (2014). The working solution consisting of 25 ml acetate buffer (300 mM), 2.5 ml TPTZ solution in 40 mM HCl, and 2.5 ml 20 mM FeCl_3_·6H_2_O solution was prepared. The mixture was incubated at 37°C. A different concentration (0.03 ml) of free/bound compounds was reacted with 0.9 ml of working solution for 30 min at 37°C. Absorbance was detected at 593 nm using a spectrophotometer. The intensity of colored compounds shows the degree of antioxidant activity (Ti et al., 2014). The bioactive compounds with a higher antioxidant activity were used to prepare nanoparticles.

#### 2.2.6 Nanoparticle preparation

To prepare oil in water nanoemulsion, free phenolic compounds (7%) were added to the oil phase containing tween 80 (25%) and kilka fish oil (68%) and stirred for 3 min at 50°C. Then, they were homogenized for 5 min using an ultraturax homogenizer (T25D; IKA) at 8870 *g* and 10°C. *Portulaca oleracea* seed gum (Khorfeh), *Trigonella foenum‐graecum* seed gum (Shanbalileh), and the mixture of both seed gums (Composite) were used to prepare three different nanoparticles. The gums were extracted as reported by Kenari et al. (2020). Briefly, the seeds were manually cleaned to remove foreign matter. Then, gums were extracted in the optimal conditions (water‐to‐seed ratio: 51:1 and pH = 5.5 at 25°C) using a hot water bath and stirring alternately. The mucilage of seeds was separated with a filter paper and dried at 70°C. The gum powders were dispersed in deionized water to achieve a total solid content of 30% and mixed using a magnetic stirrer for 15 min at ambient temperature for better dissolution. Different gum solutions were stored for 24 h in the refrigerator to complete the water absorption. The formed nanoemulsion was added slowly to various wall material solutions at a 2:10 ratio. An ultrasonic generator (UP 200S; Dr. Hielscher) was used for (6 cycles, 30 s on, and 15 s off) further size reduction in nanoemulsions. Finally, three different types of nanoparticles were prepared, which included (1) Khorfeh, (2) Shanbalileh, and (3) Composite. Nanoparticles were freeze‐dried (Zirbus Vaco5) at 0.017 mPa and −57°C for 48 h (Kenari et al., 2020; Razavi & Kenari, 2021).

#### 2.2.7 Nanoparticle properties

The properties of nanoparticles, including Z‐average size, zeta potential, and polydispersity index (PDI), were measured using a dynamic light scattering method with a Zetasizer instrument (Zetasizer‐Nano). The samples were diluted with deionized water by 1:100 ratio. Encapsulation efficiency (EE) was calculated by measuring the ratio of encapsulated phenolics to the total phenolics which were used to prepare nanoparticles (Gharehbeglou et al., 2019).

#### 2.2.8 Scanning electron microscopy images

The nanoparticles morphology was examined using a scanning electron microscope (S4800, Hitachi, Japan). Nanoparticles were attached to the sample holder with a double‐sided adhesive tape, and scanning electron microscopy (SEM) images were taken at a voltage of 15 kV and 1000 magnifications at room temperature.

#### 2.2.9 Sedimentation index and release rate of phenolic compounds

The release properties and sedimentation rate were measured using the method of Mohammadi et al. (2016). Freshly made nanoemulsions were poured into the 10‐ml glass tube to a height of 10 cm and stored at 60°C for 40 days. The height of the nanoemulsion was measured every 8 days. Sedimentation index was measured according to Equation 1:

(1) $$\text{Sedimentation}\;\text{index}\;\left(\%\right)=100\times \left(h_{i}‐h_{n}\right)/h_{i}$$

where *h*_i_ is the height of initial nanoemulsion and *h*_n_ is the height of nanoemulsion during storage.

To evaluate the release profile of phenolics, 3 g of nanoparticles was mixed with 3 g of phosphate buffer (pH = 7) and centrifuged for 60 min at 1247 *g*. The release rate of phenolics was measured according to Equation 2:

(2) $$\text{Release}\;\text{rate}\;\left(\%\right)=100\times \left(C_{t}‐C_{e}\right)/C_{t}$$

where *C*_t_ is the content of total phenolics and *C*_e_ is the content of encapsulated phenolics (Mohammadi et al., 2016).

### 2.3 Statistical analysis

Statistical analysis was performed by SPSS 20 for windows (IBM SPSS). Data were analyzed with Duncan's multiple‐range test, and all results were assessed by one‐way analysis of variance (ANOVA). A value of *p* < .05 was considered statistically significantly different. Each sample analysis was performed in triplicate.

## 3 RESULTS AND DISCUSSION

### 3.1 The content of free/bound compounds, TFC, and TPC

**FIGURE 1:**
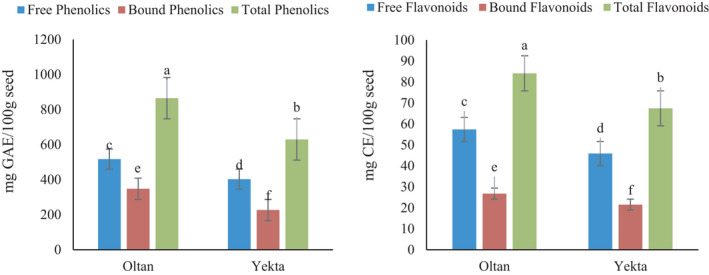
Free/bound compounds, total flavonoid content (TFC), and total phenolic content (TPC) of the sesame Oltan and Yekta varieties. Different lower‐case letters indicate statistically significant difference among means (the Duncan test, *p* < .05)

The contents of free and bound flavonoid and phenolic compounds of different sesame varieties are shown in Figure 1. As can be seen in both varieties, free flavonoid and phenolic are higher than bound compounds. The Oltan variety showed higher TFC and TPC. Bound phenolic compounds mainly have covalent bound that attach to plant cell walls. They are not generally digested by stomach acid and released in the small intestine. Flavonoids and their derivatives are the largest groups of polyphenols. Generally, free phenolic compounds form a large part of phenolics. Their content depends on the extraction method, genetic, growth, storage, sunlight, harvest, and post‐harvest conditions, and a variety of plants (Ghaderi et al., 2012; Seukep et al., 2013; Sukor et al., 2018). Ti et al. (2014) investigated the content of free and bound phenolic compounds in the bran of different rice varieties. They reported higher content of free phenolic compounds than bound type (Ti et al., 2014). A previous study by Zhou et al. (2016) showed higher free phenolics than bound in sesame. Sesamin and sesamolin are the most important free phenolic compounds extracted from sesame, while sesamol can be presented in the free or bound form. They reported the TFC in the range of 2.88–8.04 g CE/kg, which is consistent with the results of the present study (Zhou et al., 2016). The presence of phenolic compounds in sesame seed was also reported by other researchers (Abirached et al., 2020; Dravie et al., 2020; Esmaeilzadeh Kenari et al., 2014; Khan et al., 2019; Mekky et al., 2019; Mohdaly et al., 2010; Nigam et al., 2015).

### 3.2 Analysis of phenolic compounds

Table 1 illustrates the phenolic composition of the Oltan and Yekta sesame varieties. The total number of characterized compounds was 14. The major phenolic compound in sesame seed was ferulic acid, which was 15.02 and 12.41 mg/100 g DW in the Oltan and Yekta varieties, respectively. The presence of hydroxybenzoic acid, lignans (sesamol, sesamin, and sesamolin), and chlorogenic acid seed has been previously reported in sesame (Mekky et al., 2019; Ortega‐Hernández et al., 2018). The antioxidant activity of sesame seeds is related to possessing lignans and other phenolic compounds. Sesamol, sesamin, and sesamolin are the most important lignans in the sesame, which are indicated in HPLC analysis.

**TABLE 1:** Phenolic compounds characterized in the sesame Oltan and Yekta varieties (mg/100 g DW)

Number	Compounds	Molecular formula	Rt (min)	Oltan	Yakta
1	Gallic acid	C_7_H_6_O_5_	6.31	1.02^a^	0.90^b^
2	Sesamol	C_7_H_6_O_3_	7.46	1.67^a^	1.26^b^
3	Protocatechuic acid	C_7_H_6_O_4_	11.33	9.14^a^	8.17^b^
4	p‐coumaric acid	C_9_H_8_O_3_	12.12	4.74^a^	3.08^b^
5	4‐hydroxybenzoic acid	C_7_H_6_O_3_	13.88	7.11^a^	5.32^b^
6	Chlorogenic acid	C_16_H_18_O_9_	14.01	5.59^a^	4.43^b^
7	Ferulic acid	C_10_H_10_O_4_	15.35	15.02^a^	12.41^b^
8	Caffeic acid	C_9_H_8_O_4_	17.05	1.56^a^	1.03^b^
9	Catechin	C_15_H_14_O_6_	18.71	0.17^a^	0.11^b^
10	Rosmarinic acid	C_18_H_16_O_8_	19.25	4.37^a^	3.32^b^
11	Quercetin	C_15_H_10_O_7_	20.48	9.47^a^	7.12^b^
12	Sesamin	C_20_H_18_O_6_	22.34	0.16^a^	0.13^b^
13	Sesamolin	C_20_H_18_O_7_	27.90	0.08^a^	0.07^b^
14	Total			60.1	47.35

Note

Different lower‐case letters indicate significant statistical differences among means (the Duncan test, *p* < .05).

### 3.3 Antioxidant activity of compounds

The antioxidant activity of different compounds was measured by DPPH radical scavenging ability and ferric reducing antioxidant power. The first method is a simple and rapid method to measure the antioxidant activity of food substances and measures the ability of compounds to donate hydrogen atoms to quench free radicals. The second method investigates the transformation of ferric (Fe^3+^) to ferrous (Fe^2+^) iron as a measure of antioxidant activity (Dravie et al., 2020). Hence, the antioxidant ability of compounds can be examined by evaluating either its ability to transfer one electron to reduce an oxidant or its ability to donate hydrogen to quench an oxidant (Ruslan et al., 2018).

**FIGURE 2:**
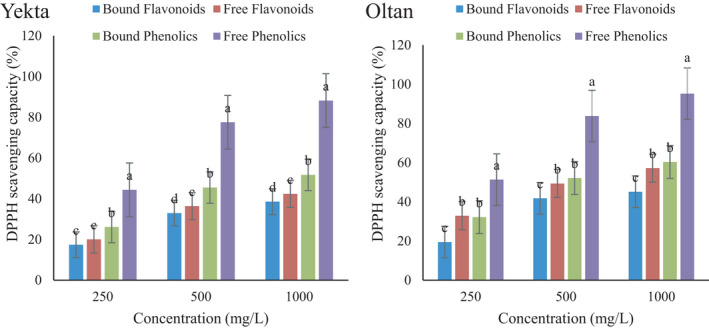
DPPH free radical scavenging activity of free/bound flavonoid and phenolic compounds of the sesame Oltan and Yekta varieties. Different lower‐case letters indicate statistically significant difference among means (the Duncan test, *p* < .05)

**FIGURE 3:**
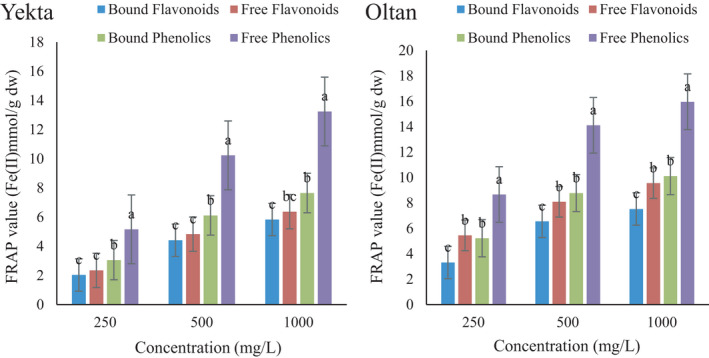
Ferric reducing antioxidant power of free/bound flavonoid and phenolic compounds of the sesame Oltan and Yekta varieties. Different lower‐case letters indicate statistically significant difference among means (the Duncan test, *p* < .05)

From the results of DPPH radical scavenging activity of different compounds in Figure 2, and the FRAP assay in Figure 3, it could be observed that antioxidant activity was increased in all compounds and that free compounds showed higher radical scavenging than bound compounds. The highest antioxidant activity was related to free phenolics. Dravie et al. (2020) reported that the acetone extract of sesame seeds demonstrated the most increased DPPH radical scavenging activity, quenching as much as 60.12% (Dravie et al., 2020).

Similar to the trend of DPPH radical scavenging activity, the antioxidant activity of different compounds increased by increasing the concentration of compounds. The antioxidant activity of plants is due to flavonoids and phenolic compounds (Park et al., 2019; Parker et al., 2003). Previous studies reported antioxidant activity and a positive correlation between phenolic content and antioxidant activity for sesame seeds (Dravie et al., 2020; Nigam et al., 2015; Othman et al., 2015; Ruslan et al., 2018). Ortega‐Hernández et al. (2018) measured the TPC and the antioxidant activity of sesame cake. They reported the highest effects on the ABTS and DPPH radicals, as well as the uppermost reducing power. They also reported a strong correlation between TPC and antioxidant activity (Ortega‐Hernández et al., 2018). Zhou et al. (2016) proved the presence of phenolic compounds, sesamin and sesamoline, in the sesame. They attributed the antioxidant activity of sesame extract to both ORAC and PSC methods (Zhou et al., 2016). Leontowicz et al. (2016) used the FRAP, ABTS, and DPPH methods to evaluate the antioxidant activity of kiwifruit extract. They reported a high correlation between the concentrations of phenolic and flavonoids contents with antioxidant activity (Leontowicz et al., 2016). A similar trend between phenolic content and antioxidant activity was found for sesame cake (Esmaeilzadeh Kenari et al., 2014; Mohdaly et al., 2011) and sesame extract (Nigam et al., 2015).

The TBHQ synthetic antioxidant at 100 ppm of concentration exhibited 90.13% and 15.45 Fe (II) mmol/g Dw in the DPPH free radical scavenging method and the FRAP assay, respectively. No statistically significant difference was observed between TBHQ and free phenolics of the Oltan variety, and therefore, these phenolic compounds were used for encapsulation.

### 3.4 Properties of nanoparticles

Dynamic light scattering (DLS) is the most common technology to determine the average size of nanoparticles, and it relies on the interaction between light and nanoparticles (Ganji & Sayyed‐Alangi, 2017). Particle size is an essential parameter in the case of emulsions and directly affects the release and absorption of encapsulated compounds (Mahalakshmi et al., 2020). The results of the particle diameter of different samples are illustrated in Table 2. All particles exhibited nanometric size in the range 236.1–417.3 nm. It can be seen that the type of wall materials has affected the size of particles. It might be due to the different viscosity and structure of gum. Mahalakshmi et al. (2020) revealed that changes in viscosity and conductivity of coating solution have a major influence on particle size (Mahalakshmi et al., 2020). The smallest and largest average size of nanoparticles belonged to particles prepared with Khorfeh and Shanbalileh wall materials and 236.1 and 680.7 nm in size, respectively. All nanoparticles had a statistically significant difference in size (*p* < .05). The formation of droplets in nanoemulsion system is the result of droplet breakage and interaction between formed droplets. It seems that diverse coatings change the size of nanoparticles by affecting the stability of nanoemulsion.

**TABLE 2:** Particle size, zeta potential, PDI, and encapsulation efficiency of phenolic compounds in different wall materials

Type of wall material	Z‐average size (nm)	Zeta potential (mV)	PDI	EE (%)
Khorfeh	236.1 ± 7.6^c^	−35.4 ± 6.5^c^	0.315^c^	61.35 ± 4.9^c^
Shanbalileh	680.7 ± 6.2^a^	−18.3 ± 3.4^a^	0.332^a^	74.49 ± 5.3^a^
Composite	417.3 ± 8.2^b^	−26.2 ± 5.7^b^	0.326^b^	66.04 ± 6.2^b^

Note

See note in Table 1.

PDI is one of the most critical features of encapsulated particles. It was used to estimate the average uniformity of nanoparticles. Given that the zeta potential shows only the surface charge of nanoparticles, the measurement of PDI is necessary. The results of PDI value of particles exhibited PDI below 0.333 (Table 2). Larger PDI corresponds to more extensive size distribution and non‐uniform droplets (Gharehbeglou et al., 2019).

Zeta potential value is an important parameter for investigating the surface electrical charge of particles and affects the rate of aggregation and flocculation of particles. It is commonly used as an indicator for the stability of emulsion systems. The results of the zeta potential of all nanoparticles showed the negative charge (Table 2). The lowest and highest absolute value of zeta potential was observed in nanoparticles prepared with Shanbalileh and Khorfeh wall materials, respectively. The negative zeta potential in all nanoparticles is related to the presence of anionic compounds of sesame's phenolics and seed gums, as well as charged functional groups and neutral structure of galactomannan. Salarbashi et al. (2019) reported negative zeta potential values in the range −20.4 to −14.8 mV for Khorfeh seed gum (Salarbashi et al., 2019). According to the matter that nanoparticles with zeta potential in the range −30 and +30 mV are unstable particles (Razavi et al., 2021; Salarbashi et al., 2019), the higher stability of nanoparticles is related to the particles with Khorfeh wall material. At a higher absolute value of zeta potential, the repulsion between gum macromolecules will be stronger and cause more stability of the nanoemulsion. The negative zeta potential of phenolics encapsulated in native seed gums such as basil (Delfanian et al., 2018), and garden cress (Taheri & Razavi, 2015), also reported in previous studies.

Phenolics are sensitive to environmental stresses such as temperature, pH, and enzymes, and therefore, encapsulation protects sensitive compounds during processes until they are released into the food or body systems (Kalušević et al., 2017). On the contrary, encapsulation prevents intense and unwanted flavors that phenolic compounds extend in food (Rezaei Savadkouhi et al., 2020). The encapsulation efficiency of nanoparticles prepared with different wall materials was higher than 61.3% (Table 2). It was reported from 0% to 95% depending on the chemical composition of wall materials (Hassani & Hasani, 2018). There was a relationship between the size of particles and encapsulation efficiency of phenolics increased from 61.35% to 74.49%, when the particle size was increased from 236.1 nm to 680.7 nm. Shanbalileh and Khorfeh seed gums had the highest and lowest encapsulation efficiency, respectively. The encapsulation efficiency was found to be dependent on the solubility of phenolics in the wall materials. Therefore, the change in the structure of wall materials significantly affects the encapsulation efficiency.

### 3.5 SEM images of nanoparticles

**FIGURE 4:**
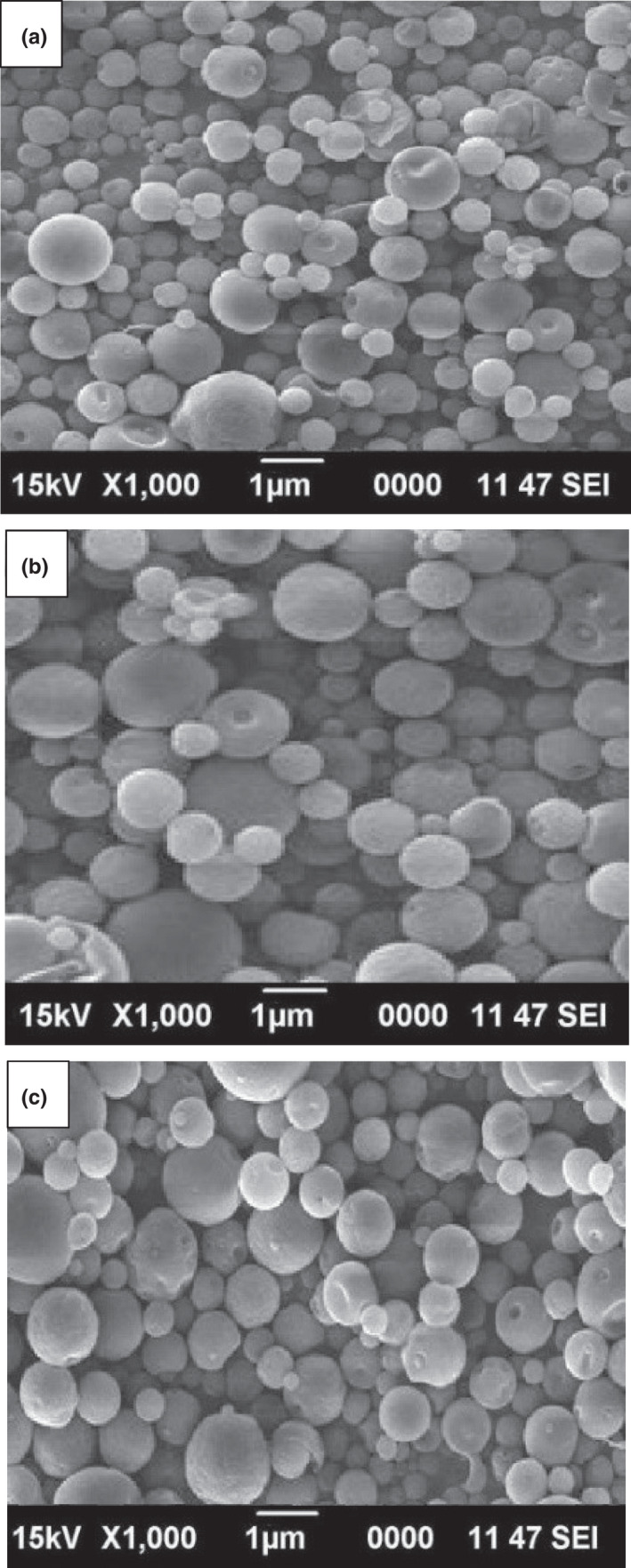
Surface electron morphology of free phenolics in Khorfeh (a), Composite (b), and Shanbalileh (c) seed gum nanoparticles

The surface evaluation of nanoparticles containing phenolics is very substantial because it must be ensured that phenolic compounds are enclosed in the wall materials. The surface electron morphology (SEM) images of different nanoparticles are displayed in Figure 4. All nanoparticles displayed a spherical‐granular structure with a smooth surface, without any shrinkage and indentation. These SEM images approve the entrapment of bioactive compounds in the core of nanoparticles and indicate the adequacy of nanoencapsulation. Generally, more sphericity and less crevice on the surface of capsules lead to better properties of nanoparticles (Kenari et al., 2020). The average particle size obtained from the SEM images was about 300–500 nm, which is a nice confirmation of the results of particle evaluation with the DLS. Various factors affect the surface properties of nanoparticles, including the nanoemulsion drying rate, core‐to‐wall material ratio, production conditions, and composition of wall materials. Given that the production conditions of nanoparticles were similar, slight differences in their surface properties are related to wall materials. Polysaccharides are highly resistant, degradable, and non‐toxic natural polymers, and due to hydrophilic properties, they are used to encapsulate compounds such as phenolics. When the drying temperature of nanoemulsion is high, they tend to lose their moisture faster, resulting in the formation of a spherical structure, confirming the efficiency of the encapsulation process and entrapment of bioactive compounds within the core (Razavi & Kenari, 2021). Therefore, according to the SEM images, it can be ensured that during the drying process, all moisture is removed from particles. The similar surface morphology was observed for gallic acid in pectin wall material (Gharehbeglou et al., 2019).

### 3.6 Release profile and sedimentation of phenolics

**FIGURE 5:**
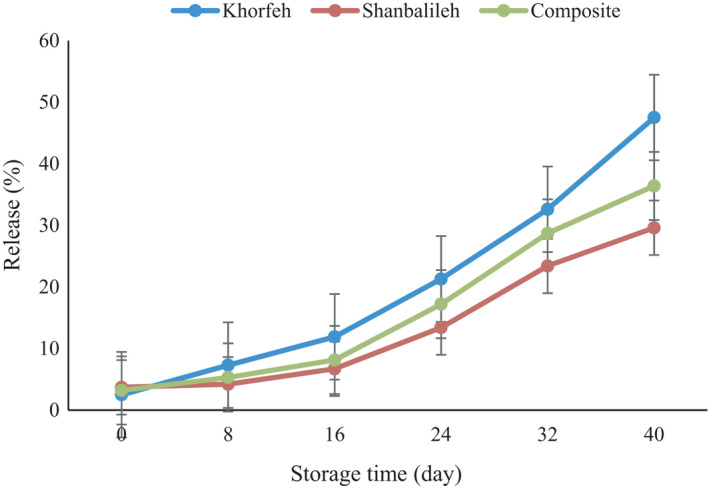
Release of phenolic compounds from nanoparticles with different wall materials

Phenolics are susceptible compounds with high reactivity, and their effects occur quickly after being added to food systems. Therefore, measuring the release rate of phenolics in food samples is very important. The release profile of phenolics is shown in Figure 5. It is observed that the release of phenolic compounds increased overtime, and the difference was statistically significant (*p* < .05). These results are in agreement with the increasing release rate of phenolic overtime as was reported for olive leaf (Mohammadi et al., 2016), grape seed (Estévez et al., 2019), bene hull (Delfanian et al., 2018), *Ferula Persica* (Estakhr et al., 2020), and Iranian golpar (Kenari et al., 2020). The higher gradual release was observed in phenolics encapsulated in Shanbalileh wall material. The nanoparticles with Khorfeh wall material showed a higher phenolic release. There is a positive correlation between particle size and phenolic release rate. The Khorfeh nanoparticles with the smallest dimension (236.1 nm) exhibited faster phenolic release. Maghamian et al. (2021) reported that the rapture of internal water droplets in the smaller size droplets is rather more. The smaller particle size increases the release rate of encapsulated compounds (Maghamian et al., 2021). Several parameters affect the release rate of bioactive compounds from nanoparticles including diffusion and distribution coefficients, trapping rate of phenolics, solubility, surface morphology, nanoparticle shape, and solubility and porosity of wall materials (Cohen & Bernstein, 2020).

**FIGURE 6:**
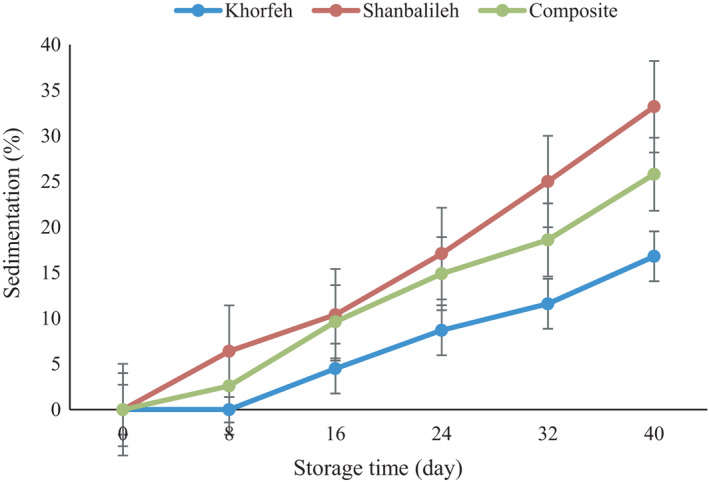
Sedimentation of phenolic compounds from nanoparticles with different wall materials

Figure 6 shows that during storage up to day 40, the sedimentation of phenolic compounds increased gradually, and a statistically significant difference has been found (*p* < .05). The nanoparticles prepared by Khorfeh seed gum were unchanged and without sedimentation up to day 8. Nishad et al. (2021) reported similar results (Nishad et al., 2021). Mohammadi et al. (2016) did not observe any sedimentation from nanoencapsulated olive leaf extract in whey protein–pectin (Mohammadi et al., 2016). The discrepancy in the sedimentation index in this study could be related to the different nature of wall materials. Reducing the droplet size caused to decrease the sedimentation index, and Khorfeh nanoparticles with smaller diameters exhibited lower sedimentation. It is in agreement with the results of Gharehbeglou et al. (2019). They revealed that smaller particles of encapsulated gallic acid were more resistant to sedimentation (Gharehbeglou et al., 2019).

## 4 CONCLUSION

Phenolics are newly discovered natural antioxidants, but if the concentration and injection method are not selected correctly, it will lead to prooxidant activities. In this study, the antioxidant potential of free/bound phenolic compounds of sesame was investigated. Both varieties of a sesame seed (Yekta and Oltan) possess a significant antioxidant activity due to flavonoid and phenolic compounds. These results provide new knowledge about different phenolic compounds in the sesame seed variety. Khorfeh and Shanbalileh seed gums exhibited excellent properties for encapsulated phenolics in terms of particle diameter, zeta potential, PDI, encapsulation efficiency, and controlled release of phenolic compounds. They can be used as a substitute for synthetic polymers and in the preparation of natural preservatives in food, which are achieved using extract encapsulation in wall materials based on native gums. These results suggest that the uptake of nanoencapsulated phenolics of sesame in composite seed gum could be potentially protected against oxidation or diverse disease.

## CONFLICT OF INTEREST

The authors declare no conflict of interest.

## AUTHOR CONTRIBUTIONS

**Reza Esmaeilzadeh Kenari:** Methodology (supporting); Resources (lead); Supervision (supporting); Writing – review & editing (equal). **Razie Razavi:** Data curation (supporting); Investigation (lead); Writing – original draft (lead); Writing – review & editing (equal).

## Data Availability

None.
